# Polycyclic Aromatic Hydrocarbon Degradation in the Sea-Surface Microlayer at Coastal Antarctica

**DOI:** 10.3389/fmicb.2022.907265

**Published:** 2022-07-14

**Authors:** Alícia Martinez-Varela, Gemma Casas, Naiara Berrojalbiz, Benjamin Piña, Jordi Dachs, Maria Vila-Costa

**Affiliations:** Department of Environmental Chemistry, Institute of Environmental Assessment and Water Research, IDAEA-CSIC, Barcelona, Spain

**Keywords:** PAH, sea-surface microlayer, hydrocarbonoclastic bacteria, Alteromonadales, PAH biodegradation, coastal Antarctica

## Abstract

As much as 400 Tg of carbon from airborne semivolatile aromatic hydrocarbons is deposited to the oceans every year, the largest identified source of anthropogenic organic carbon to the ocean. Microbial degradation is a key sink of these pollutants in surface waters, but has received little attention in polar environments. We have challenged Antarctic microbial communities from the sea-surface microlayer (SML) and the subsurface layer (SSL) with polycyclic aromatic hydrocarbons (PAHs) at environmentally relevant concentrations. PAH degradation rates and the microbial responses at both taxonomical and functional levels were assessed. Evidence for faster removal rates was observed in the SML, with rates 2.6-fold higher than in the SSL. In the SML, the highest removal rates were observed for the more hydrophobic and particle-bound PAHs. After 24 h of PAHs exposure, particle-associated bacteria in the SML showed the highest number of significant changes in their composition. These included significant enrichments of several hydrocarbonoclastic bacteria, especially the fast-growing genera *Pseudoalteromonas*, which increased their relative abundances by eightfold. Simultaneous metatranscriptomic analysis showed that the free-living fraction of SML was the most active fraction, especially for members of the order Alteromonadales, which includes *Pseudoalteromonas*. Their key role in PAHs biodegradation in polar environments should be elucidated in further studies. This study highlights the relevant role of bacterial populations inhabiting the sea-surface microlayer, especially the particle-associated habitat, as relevant bioreactors for the removal of aromatic hydrocarbons in the oceans.

## Graphical Abstract

Particle-associated bacteria inhabiting the sea-surface microlayer showed a faster capacity to degrade PAH than subsurface bacteria. 
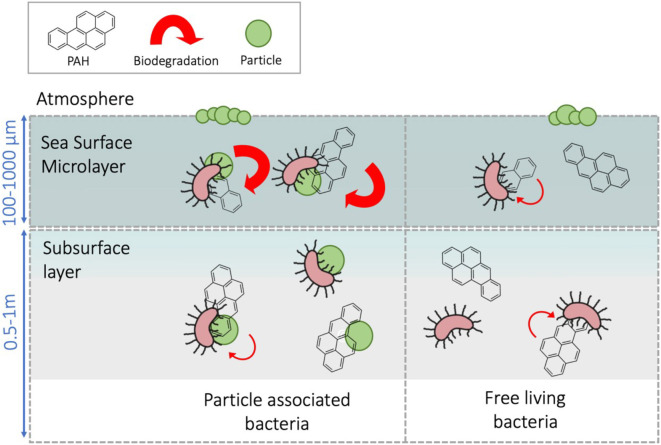

## Introduction

Polycyclic aromatic hydrocarbons (PAHs) and other semivolatile aromatic-like compounds originate from incomplete combustion of fossil fuels, oil spills, and natural processes such as oil seepage and biomass burning (Hylland, 2006; Duran and Cravo-Laureau, 2016). Semivolatile aromatic-like compounds are hydrophobic, bioaccumulative, and toxic, affecting marine organisms and posing a threat to ecosystems (Hylland, 2006; Ball and Truskewycz, 2013).

Their semivolatile nature allows them to be atmospherically transported and deposited, and in consequence, they are ubiquitous in the global ocean, even reaching remote polar regions far from their emission sources (Lohmann et al., 2009; Cabrerizo et al., 2014; González-Gaya et al., 2016; Abdel-Shafy and Mansour, 2016; Cai et al., 2016; Balmer et al., 2019; Casal et al., 2019). Quantification of atmospherically deposited PAHs into the global ocean reveals a global monthly entry of 0.09Tg C (1 Tg a year) (González-Gaya et al., 2016), which would equal four Deepwater Horizon oil spills every month (Reddy et al., 2012; González-Gaya et al., 2016). Surprisingly, only 1% of the total airborne PAHs are sequestered to the depths by the biological pump (González-Gaya et al., 2019). This and other evidences suggest that a major fraction of airborne aromatic hydrocarbons is removed at the surface ocean by microbial degradation and, to a lesser extent, by photo-degradation (de Bruyn et al., 2012; González-Gaya et al., 2019).

In oil-contaminated environments, microbial degradation is the major PAHs removal process, mediated by hydrocarbonoclastic bacteria (HCB, specialist and facultative hydrocarbon degraders) (Head et al., 2006; Dong et al., 2015; Crisafi et al., 2016; Berry and Gutierrez, 2017; Gontikaki et al., 2018). Highly polluted sites, such as oil spill-affected areas from temperate and polar regions, have provided the current knowledge of the biogeochemical and molecular basis for the biodegradation of PAHs (Lors et al., 2012; Dombrowski et al., 2016; Ghosal et al., 2016; Gupte et al., 2016; Joye et al., 2018). However, *in situ* measurements of PAHs degradation rates are comparatively scarce, and only few studies have focused on the microbial responses to airborne PAHs, semivolatile aromatic-like compounds, and other organic pollutants at environmentally relevant concentrations (Cerro-Gálvez et al., 2019; González-Gaya et al., 2019; Vila-Costa et al., 2019; Martinez-Varela et al., 2021).

The sea-surface microlayer (SML) is the thin interface between the ocean and the atmosphere, operationally defined as the top 100–1,000 μm of the marine water column (Liss et al., 2005). The active air–sea exchange and mixing dynamics of particles, gases, and water through the SML contribute to the enrichment of amphiphilic, hydrophobic, surfactant-like compounds and an array of miscellaneous organic molecules at the SML (Calace et al., 2004; Sabbaghzadeh et al., 2017; Uning et al., 2018), including atmospherically deposited PAHs (Wurl and Obbard, 2004; Cincinelli et al., 2005; Stortini et al., 2009) and other organic pollutants such as polychlorinated biphenyls (Fuoco et al., 2005; García-Flor et al., 2005) and perfluoroalkyl substances (Ju et al., 2008; Casas et al., 2020). Bacteria inhabiting the SML are known as bacterioneuston (Naumann, 1917) and are exposed to unique physicochemical properties and harsh environmental stressors. Bacterioneuston participates in air–sea interactions influencing key biogeochemical and climate-related processes (Kuznetsova and Lee, 2001; Brooks et al., 2009; Nakajima et al., 2013). However, very few works addressed the interaction of bacterioneuston and pollution by PAHs and other airborne organic contaminants at low concentrations. For instance, Coelho et al. (2011) explored the potential of bacterioneuston strains to degrade PAHs by isolating HCB from the SML of a chronically oil-polluted estuarine (Coelho et al., 2011). Martinez-Varela et al. (2020) found strong correlations between the enrichment of perfluoroalkyl substances (one of the ubiquitous constituents within anthropogenic dissolved organic carbon (ADOC)) and ADOC-degrading strains at the Antarctic SML (Martinez-Varela et al., 2020). Unfortunately, analyses of responses to PAHs and other ADOC compounds are still scarce for the marine environment and absent in some compartments such as the SML.

In the Southern Ocean, atmospheric diffusive loadings and snow deposition represent the major source of PAHs into the ocean (Cabrerizo et al., 2014; Casal et al., 2019). Several field measurements from Antarctica (Cincinelli et al., 2001; Guitart et al., 2004; Fuoco et al., 2005; Stortini et al., 2009) and other marine regions (Guitart et al., 2007; Lim et al., 2007; Huang et al., 2020) showed that PAHs accumulate at the SML due to their hydrophobic nature, reporting enrichment factors up to eightfold (Cincinelli et al., 2005; Fuoco et al., 2005; Stortini et al., 2009). Previous studies have shown than particle-associated (PA) bacteria differ from the free-living (FL) fraction, at both taxonomical and functional levels (Smith et al., 2013; Hu et al., 2020). Whether or not high concentrations of PAHs sorbed onto/into particles stimulate those bacterial communities better suited for PAHs degradation remains unknown.

The goal of this study was to characterize and compare the PAHs degradation capacity of two fractions, namely, PA and FL, of the bacterial communities present in the two adjacent habitats of the surface ocean, namely, SML and subsurface layer (SSL), in coastal Antarctica. We exposed each Antarctic bacterial community to environmental concentrations of PAHs for 24 h, measured PAHs degradation rates, and assessed the responses at both taxonomical and functional levels by means of 16S rRNA amplicon sequencing and metatranscriptomics. This approach allowed answering whether or not SML is a neglected hot spot of PAHs degradation in Antarctic waters and elucidating the main players in biodegradation.

## Materials and Methods

### Sampling and Site Description

Seawater from the SML and SSL was collected over the mid-austral summer (February 21, 2018) at the South Bay of Livingston Island (South Shetlands, Antarctica). SML and SSL sampling was conducted from a rigid inflatable boat at 62°38.346′S, 60°23.912′W (Supplementary Figure 1). The SML was sampled with a glass plate SML sampler. This sampling method was first described by Harvey and Burzell (1972) and has been used in a number of studies for sampling organic matter, microorganisms, surfactants, and organic pollutants in the SML (Knulst et al., 2003; García-Flor et al., 2005; Ju et al., 2008; Casas et al., 2020; Martinez-Varela et al., 2020). The device was sterilized with 70% v/v ethanol followed by Milli-Q water and rinsed three times with seawater from the sampling site. The plate (40 × 30 cm) was inserted vertically into the water and then withdrawn slowly allowing the SML adhering onto the glass plate. The glass plate containing the SML sample was then wiped between two Teflon (PTFE) plates draining the sample into 1-L 400°C pre-baked glass bottles. This process was repeated until obtaining a volume of 6 L of SML water. Subsurface layer seawater samples were collected in 2-L PP bottles by submerging the bottles and opening it at 0.5 m depth. Simultaneously, physicochemical parameters were measured at the sampling station with a CTD probe (Supplementary Table 1). Meteorological conditions during the sampling event were measured by the Meteorological Spanish Agency at the meteorological station located in South Bay of Livingston Island.

### Experimental Setup With Natural Communities From the Subsurface Layer and the Sea-Surface Microlayer

Three hours prior to the surface seawater sampling, a mixture of 16 parent PAHs (PAH-mix 9, Dr. Ehrenstorfer, see section on PAHs analysis) was added to 1-L pre-cleaned and 400°C pre-baked empty glass bottles. The same process was followed in the control bottles, where the same volume of solvent without PAHs spike was added. Then, the solvent (cyclohexane) was allowed to evaporate in treatment and control bottles to avoid any toxic effects derived from the presence of solvent. Nominal individual PAH concentrations in the treatments were 200 ng L^–1^. 0.7 L of seawater from SML and SSL was dispensed into the experimental bottles. Two replicates were performed for each unique experimental condition. Abiotic controls were run with HPLC-grade water with two replicates and were incubated, sampled, and analyzed following the same sample procedure than the alive treatments. The experimental incubations were carried out at an outdoor deck of the Spanish Antarctic Base facilities in Livingston Island (62°39′43″S, 60°23′17″W). Experiment bottles were incubated during 24 h in the dark, at an *in situ* temperature between 1 and 2°C.

### Bacterial Abundance

Prokaryotic cell abundance was estimated by flow cytometry as described elsewhere (Falcioni et al., 2008). In brief, fixed samples (0.4 ml) for heterotrophic non-pigmented total bacteria enumeration were stained with 4 μl of a 10 × SG1 (Molecular Probes) solution (final dilution, 1:1,000 [vol/vol]) for 10 min and run through a FACSCalibur flow cytometer at a low speed (15 μl min^–1^), with fluorescent microspheres as an internal standard (yellow–green 0.92-μm Polysciences latex beads [10^6^ml^–1^]). Bacteria were detected in a dot plot of side scatter versus green fluorescence (FL1), and a population concentration was estimated with CellQuest and PaintAGate software (Becton Dickinson, Palo Alto, CA, United States).

### Nucleic Acids Extraction and Sequencing

At the beginning of the experiment and after 24 h of PAHs addition, 0.69 L of seawater of each layer was pre-filtered through a 3-μm-pore-size 47-mm-diameter polytetrafluoroethylene filter to collect the particle-associated bacteria (PA; > 3.0 μm). Cells in the filtrate were then collected on a 0.2-μm-pore-size 47-mm-diameter polytetrafluoroethylene filter to obtain the free-living fraction (FL; 0.2–3.0 μm), using a peristaltic pump with a flow of < 50 mL min^–1^. The duration of the filtration step was no longer than 15 min to minimize RNA degradation. Each filter was cut into two halves, one being placed in 1 ml RNAlater (Sigma-Aldrich, St. Louis, MO, United States) and the other one in 1 ml lysis buffer (50 mM Tris–HCl, 40 mM EDTA, 0.75 M sucrose), and stored at –20°C to preserve RNA and DNA, respectively. DNA was extracted as described in Vila-Costa et al. (2019). Total RNA was extracted, DNA removed, rRNA depleted, and mRNA enriched by amplification as described in Poretsky et al. (2010) with the only modification that total RNA was extracted with mirVana isolation kit (Ambion), after removing the storage reagent by centrifugation.

Partial bacterial 16S gene fragments of both DNA and cDNA were amplified using primers 515F-Y (5′-GTGYCAGCMGCCGCGGTAA) and 926R (5′-CCGYCAATTYMTTTRAGTTT) (Parada et al., 2016) plus adaptors for Illumina MiSeq sequencing. The PCR mixture was thermocycled at 95°C for 3 min, 30 cycles at 95°C for 45 s, 50°C for 45 s, and 68°C for 90 s, followed by a final extension of 5 min at 68°C. PCR amplicon sizes were checked in Tris–acetate–EDTA (TAE) agarose gels. Illumina MiSeq sequencing was performed at the Pompeu Fabra University Sequencing Service.

The complete nucleotide and transcript sequence dataset generated and analyzed in this study was deposited in the sequence read archive (SRA) under the bioproject accession nos. PRJNA739708 and SUB9892364.

### Dissolved Phase Concentrations of Polycyclic Aromatic Hydrocarbons

Concentrations of PAHs were measured in the dissolved phase at the beginning (T0) of the experiment and at the end (T24) of the incubation using the filtrate obtained after sampling DNA/RNA material (Supplementary Table 2), using the same filtration procedure for treatments and controls. SSL and SML water in treatments and controls and HPLC water from abiotic controls were concentrated on Bond Elut C18 cartridges (500 mg, Agilent), after filtration, using a Baker vacuum system, adapting a previously described methodology (Berrojalbiz et al., 2009). After drying the cartridges under vacuum, these were air-tight wrapped and kept at −20°C until their further analysis in the laboratory. Once in the ultra-clean laboratory in Barcelona, PAHs were extracted with hexane/dichloromethane (1:1, v/v). Then, the extract was concentrated to 0.5 mL by vacuum rotary evaporation, transferred to vials, and further evaporated to 400 μL under a gentle nitrogen stream. Before quantification, per-deuterated internal standards were added to vials. PAHs analyses were performed by gas chromatography coupled with mass spectrometry (GC–MS). The targeted PAHs were fluorene, anthracene, phenanthrene, pyrene, fluoranthene, chrysene, benzo(a)anthracene, benzo(b)fluoranthene, benzo(k)fluoranthene, benzo(a)pyrene, dibenzo(a,h)anthracene, indeno(1,2,3-cd)pyrene, and benzo(ghi)perylene. Even though naphthalene, acenaphthene, and acenaphthylene were present in the PAHs mix used, they were excluded from the study as our analytical procedure is not appropriate for a quantitative analysis of these volatile PAHs. Surrogate standards were added before extraction to calculate the target analyte recoveries to ensure the quality of the analytical procedure. An internal standard procedure was used to quantify the samples.

The PAHs removal rates were calculated by the difference between initial and final time point concentrations. In all treatments, concentrations were above limits of quantification. During the time course of the incubations, the PAHs added in seawater undergo partitioning to the organic matter pools due to their hydrophobicity. Then, it is possible that part of the apparent decrease of concentrations is due to partitioning to cells (bacteria and phytoplankton). The extent of this potential artifact of the removal rates estimates was assessed assuming that the microorganism–water bioconcentration factor was proportional to the hydrophobicity of the chemical, as given by the octanol–water partition constant (K_OW_).


(1) $$BCF=\frac{C_{M}}{C_{W}}={\text{f}}_{OM}\frac{K_{OW}}{\delta }$$

where C_M_ and C_W_ are the PAHs concentrations in the microorganism and water, respectively, f_OM_ is the fraction of organic matter, and δ is the density of octanol. Such dependence of BCF on K_OW_ has been reported before for phytoplankton and bacteria (Del Vento and Dachs, 2002; Berrojalbiz et al., 2009; Wang and Kelly, 2018). This allowed estimating the concentration of individual PAHs in microorganism (bacteria and phytoplankton). The biomass of bacteria was estimated from the cell counts obtained from flow cytometry and assuming a carbon content of 20 fgC cell^–1^ (Lee and Fuhrman, 1987). The biomass of phytoplankton was obtained by concurrent plankton sampling with vertical tows of a 50-μm mesh net as explained elsewhere (Casal et al., 2018). Similarly, sorption to DOC was estimated by assuming that the DOC–water partition coefficient was 0.1K_OW_, as commonly done (González-Gaya et al., 2016), and considering a DOC content of 60 μM which is the average value in Antarctic seawater (Doval et al., 2002).

Benchmarking approach (Mclachlan et al., 2017) was performed using phenanthrene as normalizing chemical because it did not show significant differences in removal rates between layers. Benchmarking approach is commonly used to correct for artifacts in chemical concentrations when working with low volumes of water and glass bottles (Cerro-Gálvez et al., 2020).

### Bioinformatics

Spurious sequences and primers were trimmed using Cutadapt v.1.16 (Martin, 2011). The cutoff before a base would be trimmed from either end of the read was set with a minimum Phred quality score of 20 (1% error rate), and primers and Illumina adapters were removed with a minimum overlap of 10 base pairs. DADA2 v1.4 was used to differentiate the 16S V4-5 amplicon sequence variants (ASVs) and remove chimeras (parameters: maxN = 0, maxEE = 2,4, truncLen = 220,200) (Callahan et al., 2016). DADA2 resolves ASVs by modeling the errors in Illumina-sequenced amplicon reads. The approach is threshold-free, inferring exact variants up to one nucleotide difference using the quality score distribution in a probability model. A taxonomic assignment of the ASVs was performed with the SILVA algorithm classifier against SILVA database release 138 (Quast et al., 2013). ASVs classified as mitochondria or chloroplast were removed. The final ASV table contained 23 samples (see details in Supplementary Table 4). The entire sample set yielded a total of unique 1096 amplicon sequence variants (ASVs) from 16S rRNA gene V4-5 fragments. The average number of sequences per samples was 56215.08 ± 34382.35 and 30024 ± 9982.27 for FL and PA fraction, respectively, with a minimum sequencing depth of 11,664 sequences. The maximum and minimum number of unique ASVs per sample was 253 and 71, respectively, averaging 147.39 ± 45.75 of unique bacterial ASV per sample. Rarefaction was done to the minimum sequencing depth with rrarefy() function from package vegan v1.4-4″ in R environment (Oksanen et al., 2020). Archaea accounted for < 1% of the total pool of reads and were discarded from the analyses.

For the metatranscriptomes, cDNA sequences were quality trimmed with Cutadapt (Martin, 2011) and Sickle (Joshi and Fass, 2011) and internal standards and any remaining stable RNA was quantified and removed using the Bowtie2 mapping program against the internal standard sequences and an in-house database of marine bacterial stable RNA sequences (rRNA and tRNA), respectively. Subsequently, high-quality reads were *de novo* assembled using the MEGAHIT program (v1.1.2) (Li et al., 2016) with default parameters. Open reading frame (ORF) prediction was performed using Prodigal (Hyatt et al., 2010). Contigs were then aligned to the NCBI RefSeq database (downloaded October 2016) using the Diamond aligner (v0.9.24) (Buchfink et al., 2015) in blastx mode with default parameters. Functional SEED classification and taxonomic affiliation were assigned with MEGAN 6.7.3 (Huson et al., 2016). Transcripts were mapped to the open reading frames (ORFs) using bowtie2 (v2.3.5.1) and quantified using SAMtools (v1.9). Data were then exported for further analysis in R/tidyverse. Relative raw gene counts were normalized to library sizes (Supplementary Table 5).

The expression of specific PAH-degrading genes within the metatranscriptomes was searched using a HMMER search against Pfam HMMER PAH degradation profiles. The list of the specific Pfam profiles used is shown in Figure 4 and Supplementary Table 6 and was obtained from “MetaCyc” (Caspi et al., 2020) and downloaded from “Pfam” database (Mistry et al., 2021).

**FIGURE 1:**
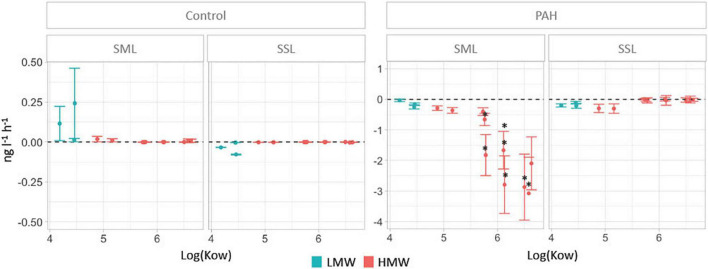
PAH removal rates in the sea-surface microlayer (SML) and the subsurface layer (SSL) for treatments and controls calculated as the difference of concentrations between time 0 and 24 h (units in ng L^–1^ h^–1^). Significant differences between layers (Mann–Whitney test, *P* < 0.05) are labeled with an asterisk.

**FIGURE 2:**
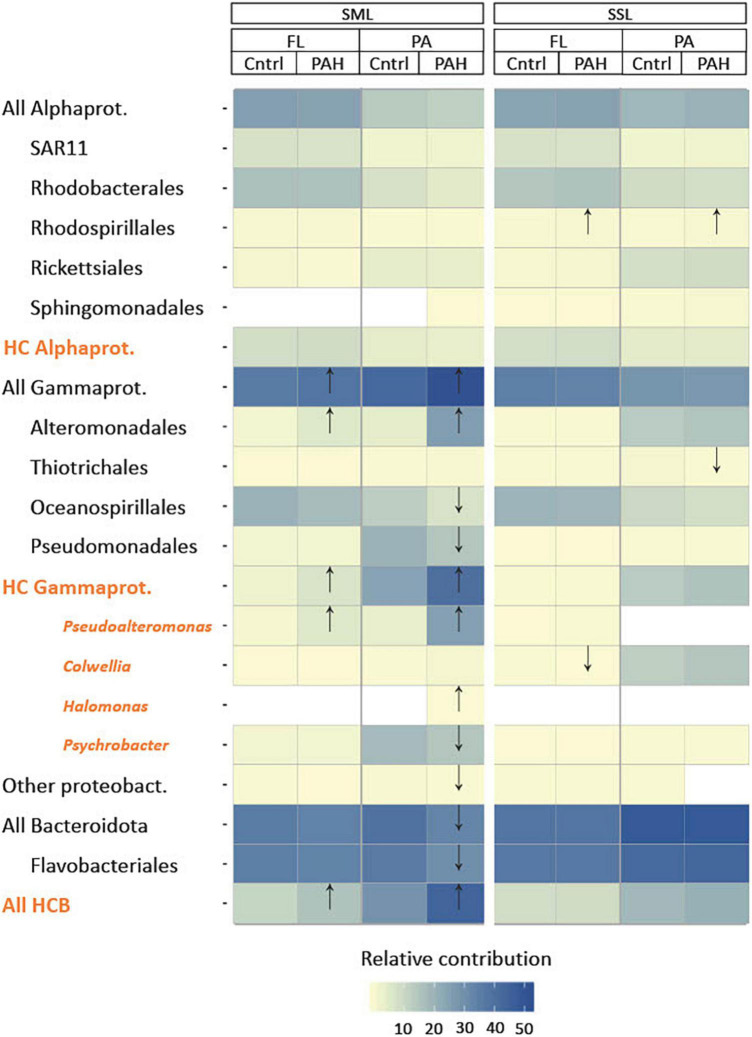
Characterization of community structure after 24 h of PAH exposure in the sea-surface microlayer (SML) and subsurface layer (SSL) for particle-associated (PA) and free-living (FL) bacterial fractions based on 16S rRNA gene amplicon sequencing. Statistical differences between controls and PAH treatments were detected by *t*-test and labeled with an arrow in the figure. Hydrocarbonoclastic bacteria (HCB) (in orange) as listed in Supplementary Table 7. Notice that general taxonomical groups (in black) include HCB.

**FIGURE 3:**
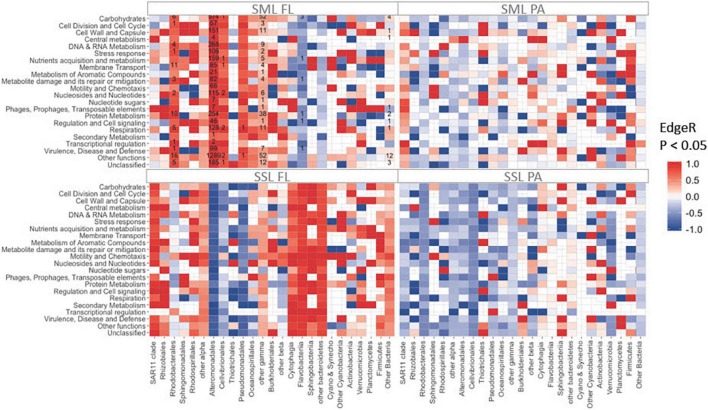
Total number of significantly enriched (in red) or depleted (in blue) transcripts detected by EdgeR (FDR < 0.05) between PAH treatments and controls metatranscriptomes. Counts are indicated inside each tile. Rows correspond to SEED categories (SML, sea-surface microlayer; SSL, subsurface layer; PA, particle-associated; FL, free-living).

**FIGURE 4:**
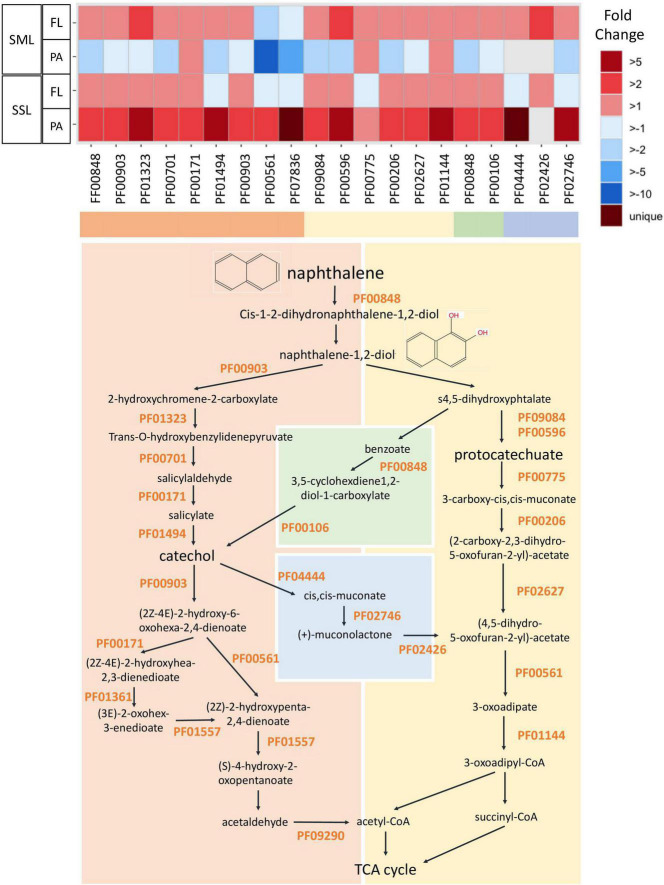
Fold change in expression of genes involved in PAH degradation between PAH treatments and controls measured by metatranscriptomics. PAH degradation pathway is represented for the naphthalene (model and simplest PAH compound). The list of the specific Pfam profiles involved in PAH degradation is shown in Supplementary Table 4 (SML, sea-surface microlayer; SSL, subsurface layer; PA, particle-associated; FL, free-living).

### Identification of Hydrocarbonoclastic Bacterial Genera

A list of hydrocarbonoclastic bacterial (HCB) genera was retrieved from the literature, including genera either collected from hydrocarbon-polluted environments, observed to have stimulated growth following hydrocarbon exposure or showing hydrocarbon catabolic activity, from both isolates and marine environments [after metagenome-assembled genome (MAGS) reconstruction] (Lozada et al., 2014; Karthikeyan et al., 2020).

The detection of specific HCB genera was performed by filtering at genus level all those ASVs and transcripts with taxonomical affiliation matching to those targeted HCB groups (Supplementary Table 7). Although in some cases it might not be hold, HCB-ASV and transcripts within the same genus were assumed to share similar metabolism as it has been observed for most HCB taxa by Gutierrez et al. (2013b).

### Statistical Analyses

R software was used to perform the different statistical analyses. Significant differences in removal rates between layers were tested with the non-parametric Mann–Whitney test (unpaired Wilcoxon’s rank test, from package stats v. 3.6.2, *P* ≤ 0.05). Community composition significant differences between layers were tested with a permutation analysis of variance (PERMANOVA, with function Adonis from package vegan v1.4-4). Fold changes of specific taxa were calculated for the relative values between layers (SML vs. SSL) and between treatments (PAHs exposed vs. controls) using the function fold change (package gtools v. 3.9.2) (R Core Team, 2019). Significant differences of specific taxa between treatments were tested with a paired two-sample *t*-test (using the “t.test” function from package stats v. 3.6.2, *P* ≤ 0.05). Analysis of differentially expressed genes was performed with the “EdgeR” package v. 3.36.0 (Robinson et al., 2009). In brief, metatranscriptomic data and the design matrix of the experiment were fitted into a negative binomial generalized linear model, and dispersion estimates from the read counts across all samples were obtained. Differentially expressed genes between experimental groups were determined by using likelihood ratio test. Significance is tested through data randomizations with threshold set by the false discovery rate correction, FDR ≤ 0.05. Further graphs were plotted using the package ggplot v. 3.3.5, also in R environment (version 4.1.1.) (Wickham, 2009).

## Results and Discussion

### Characterization of Initial Conditions at the Sea-Surface Microlayer and Subsurface Layer

Initial seawater physicochemical and meteorological conditions are summarized in Supplementary Table 1. Sea-surface temperature, wind speed, and radiation conditions (1.9°C, 2.4 ms^–1^, and 5.7 Wm^2^, respectively) were favorable for the formation of SML (Supplementary Table 1). The concentrations of nutrients confirm a non-nutrient limited system, consistent with the prevalent upwelling in the region during the austral summer (Garcia et al., 2013) (Supplementary Table 8). Total dissolved phase PAH concentrations were 0.74 ± 0.14 ng L^–1^ in the SML and 3.63 ± 0.19 ng L^–1^ in the SSL (Supplementary Table 2), in agreement with previous studies carried out in South Bay (Casal et al., 2018).

Analysis of bacterial composition by 16S rRNA gene amplicon sequencing showed similar initial bacterial populations in both layers, although Pseudomonadales were significantly enriched at the SML (*t*-test, *P* < 0.02, Supplementary Figure 3). Microbial community composition in the FL fraction was significantly different than that in the PA fraction (PERMANOVA *P* = 0.03, Supplementary Figure 4 and Supplementary Table 9), consistent with observations from other studies (Eloe et al., 2011; Rieck et al., 2015; Roth Rosenberg et al., 2021). The SAR11 clade, Rhodobacterales and Oceanospirillales, while dominant in both habitats, showed a significant preference for the FL life style (*t*-test, *P* < 0.03, Supplementary Table 9).

The PAH-degrading potential of each community was estimated by quantifying the relative abundance of HCB at the initial conditions. The contribution of HCB to the total pool of 16S rRNA gene was significantly higher in the PA than in the FL fractions in both layers (*t*-test, *P* < 0.04 for the SML and *P* < 0.01 for the SSL, Supplementary Tables 7, 9 and Supplementary Figure 5). *Sulfitobacter* was dominant within the HCB pool at initial conditions, accounting for an average of 4.4 ± 0.9% of total 16S rRNA genes. Some strains were almost specific of each layer: *Pseudoalteromonas* and *Psychrobacter* strains, known to be highly resistant to solar radiation (Agogué et al., 2005; Santos et al., 2012), were abundant within the HCB pool in the SML and almost absent in the SSL, while *Colwellia* predominated only at the PA from the SSL. A previous work has shown an enrichment of pollutant degraders in the surface microlayer from South Bay (Martinez-Varela et al., 2020) for the same austral summer, even though with an important day-to-day variability.

### Polycyclic Aromatic Hydrocarbons Degradation Rates at the Sea-Surface Microlayer and Subsurface Layer

The degradation of PAHs in the SML and SSL was measured in short-term experiments (24 h) with treatments spiked with PAHs, but with *in situ* concentrations in the controls. PAH concentrations measured in PAH treatments and in both biotic and abiotic controls at time 0 and 24 h are shown in Supplementary Table 2. The PAHs-spiked concentrations are in the range of the natural variability of PAHs in the marine environment and are below the concentrations of total semivolatile aromatic-like compounds in the open ocean (González-Gaya et al., 2019). Thus, the assessment of PAHs removal and changes in bacterial composition and activity were measured under environmentally relevant conditions.

Significantly higher PAHs removal rates were measured in the SML than in the SSL for chrysene, benzo(a)anthracene, benzo(a)pyrene, benzo(k)fluoranthene, benzo(b)fluoranthene, dibenzo(a,h)anthracene, indeno(1,2,3-cd)pyrene, and the sum of HMW (Figure 1, Mann–Whitney test, *P* < 0.04). At the SML, the removal rates of individual high molecular weight PAHs compounds (HMW, PAHs with four or more fused rings) ranged from 0.29 ± 0.08 ng L^–1^ h^–1^ to 3.08 ± 1.2 ng L^–1^ h^–1^, averaging 1.6 ng L^–1^ h^–1^. Low molecular weight (LMW) PAHs at the SML showed significantly lower biodegradation rates than HMW PAHs, ranging from 0.03 ± 0.04 ng L^–1^ h^–1^ to 0.25 ± 0.07 ng L^–1^ h^–1^, averaging 0.14 ng L^–1^ h^–1^ (Mann–Whitney test, *P* < 0.04). These removal rates contrast with those at the SSL, where HMW PAHs remained mostly unchanged, while LMW PAHs were removed at an average rate of 0.17 ± 0.03 ng L^–1^ h^–1^, comparable to the observed in the SML (Figure 1). Differences in the removal rates for concentrations corrected by benchmarking (see section “Materials and Methods”) confirmed that they were significant for benzo(a)anthracene, benzo(a)pyrene, benzo(b)fluoranthene, benzo(ghi)perylene, benzo(k)fluoranthene, chrysene, dibenzo(a,h)anthracene, indeno(1,2,3-cd)pyrene, and the sum of all HMW (Supplementary Figure 2).

Removal rates of PAHs at the SML showed an inverse correlation with the K_*OW*_ values for the different PAHs, a trend not observed in the SSL (Figure 1). These rates were measured in the dissolved phase concentrations, so the removal of PAHs could potentially be caused either by the abiotic process of diffusive partitioning of PAHs to the particulate organic carbon pool (POC, that is, mostly bacteria and phytoplankton), or due to biodegradation. To distinguish between both processes, partitioning was modeled from field data (see section “Materials and Methods” for details). The more hydrophobic the PAHs is, the more the chemical bioconcentrates in the cells (Del Vento and Dachs, 2002). There is the possibility, then, that the decrease of HMW PAHs at SML simply reflected their sorption to the POC pool. However, the fraction of PAHs retained in bacteria and phytoplankton was estimated to range between 0.1% for LMW PAHs and up to 9% for the most hydrophobic HMW PAHs. Similar estimates were found due to sorption to DOC. Both microorganism abundance and DOC cannot vary remarkably over a 24-h period and thus cannot account for the strong decrease of HMW PAHs. In any case, the potential decrease to sorption to changing organic matter pools is remarkably lower than the average decrease of 52.2 ± 1% for HMW PAHs at the SML, strongly suggesting that biodegradation was the main removal process. Direct photo-degradation could not occur as experiments were run under dark conditions.

The fact that the removal rates were significantly higher for HMW PAHs than for LMW PAHs (*t*-test, *P* = 0.04) suggests that biodegradation mostly occurred in the particle phase, that is, in the PA habitat. This is consistent with the proposed fast water–bacteria partitioning model (Axelman et al., 1997; Del Vento and Dachs, 2002), in which diffusion of PAHs from the dissolved phase is much higher than their rate of degradation either inside or at the vicinity of the cell surface. In fact, in the environment, BCF values are kept constant (close to steady state), and this explains the fact that the removal of hydrophobic PAHs was mirrored by the dissolved phase concentrations of PAHs. HMW PAHs are highly hydrophobic and thus are mainly present in the particulate organic carbon, mainly bacteria, phytoplankton, and detritus.

Our results are at variance with previous experimental data using cultures of HCB retrieved from polluted environments, which showed similar 24-h biodegradation rates for LWM and HMW PAHs, although they were performed under different conditions from those present in the maritime Antarctica (Kanaly and Harayama, 2010; Huang et al., 2016; Umar et al., 2017; Khatoon and Malik, 2019; Medić et al., 2020). In a polar environment, sub-ice bacterial communities have the capacity to remove up to 95% of LMW and HMW PAHs from a mesocosm, but after a longer period (15 days) (Garneau et al., 2016) than in our experiment (1 day). Taken together, these data illustrate the remarkable capacity of SML communities to quickly degrade PAHs at low temperatures.

### Changes in Community Composition Due to Exposure to Polycyclic Aromatic Hydrocarbons

Exposure to PAHs for 24 h resulted in significant changes in the SML bacterial community, particularly for the PA bacterial fraction, whereas the SSL populations remained relatively unaltered (Figure 2). HCB genera appeared particularly enriched in the PAHs-exposed SML populations, especially in the PA fraction, consistently with HMW PAHs hydrophobicity facilitating the adsorption to the POC present in the SML (Figure 2). More specifically, the facultative HCB *Pseudoalteromonas* showed the highest increase in relative abundance between control and PAHs treatment from 3.7% to 25.6 ± 0.3% (for the PA) and from 0.8 ± 0.2% to 5.8 ± 0.9% (for the FL) after 24 h of exposure (Supplementary Table 9). This increase suggests that even subtle PAH concentrations can trigger the exponential growth of low abundant taxa, becoming key players for PAH–microbiome interaction, as observed both in polar and in temperate seas (Sauret et al., 2014; Cerro-Gálvez et al., 2019; Martinez-Varela et al., 2021). *Pseudoalteromonas* species are early responders following marine oil spills (Dubinsky et al., 2013; Krolicka et al., 2017; Gutierrez et al., 2018), as well as in PAHs-enriched mesocosms experiments at low temperatures (Dong et al., 2015; Krolicka et al., 2017). Their nutritional preference toward PAHs has been described in several studies (Hedlund and Staley, 2006; Hazen et al., 2010; Jin et al., 2016). *Pseudoalteromonas* and other Alteromonadales strains, such as *Glaciecola, Colwellia*, and *Alteromonas*, produce exopolymeric polysaccharide substances (EPS) with amphiphilic properties (Gutierrez et al., 2013a; Dang et al., 2016; Roca et al., 2016; Mapelli et al., 2017; Supplementary Table 7) which may increase PAHs solubility and bioavailability by increasing the interfacial surface between hydrophobic substrates and bacteria (Gutierrez et al., 2013a; Quigg et al., 2016). EPS also provide a physical structure facilitating faster transformation steps in PA communities, as observed with marine oil snow (Hatcher et al., 2018). Other than the direct responses of bacteria to PAHs consumption and exposure, enrichments or depletions of some taxa after PAHs addition might result from indirect negative effects of PAHs on phytoplankton and grazers, thus affecting the mortality of certain groups (Barata et al., 2005; Fernández-Pinos et al., 2017).

Other HCB groups such as HC Bacteroidia, and the cold-adapted hydrocarbon-degrading genus *Colwellia*, showed fold changes above 2 in the SML when exposed to PAHs (Supplementary Table 10). Simultaneously, at the SML PA fraction, significant decreases in their relative abundances were observed for Oceanospirillales, Pseudomonadales, All Bacteroidota, Flavobacteriales (as a whole class), and the HCB *Psychrobacter*. This suggests not all taxa were favored by PAHs exposure.

At the SSL, Rhodospirillales, including different marine strains from genera *Thalassospira*, capable of degrading PAHs (Kodama et al., 2008; Kim and Kwon, 2010; Zhao et al., 2010), increased significantly their relative contribution in both fractions after PAHs exposure. Conversely, *Colwellia sp.* and Thiotrichales decreased their relative abundance at the PAHs-exposed community at FL and PA, respectively.

### Changes in Gene Expression Profiles After 24 h of Polycyclic Aromatic Hydrocarbons Exposure

The snapshot of activities of the different groups of bacteria 24 h after PAHs exposure was assessed by analyzing gene expression profiles by metatranscriptomic approaches. Overall, the highest number of significant changes at the PAHs-exposed communities was observed at the FL SML [Figure 3, EdgeR (Robinson et al., 2009) FDR < 0.05]. In this fraction, most of the functional enrichments of differentially expressed genes (DEG) were mainly associated with Alteromonadales, but also with Rhodobacterales, Cellvibrionales, and other Gammaproteobacteria (Figure 3). Notably, Alteromonadales showed significant increases in expression levels at the PAHs-exposed community for all functional categories, including metabolism of aromatic compounds, supporting its pivotal key role in PAH degradation. This was consistent with the *Pseudoalteromonas* major role in explaining 16S community shift responses in the SML.

Polycyclic aromatic hydrocarbon degradation genes were tracked based on the Pfam profiles integrated in the degradation pathway of the model PAH naphthalene (Figure 4). The ring hydroxylating dioxygenase (RHD), a catalyzer complex of the initiating PAH dihydroxylation step (PF00848), was found enriched in all PAHs-exposed communities except for the SML PA (Figure 4). However, when looking into the transcripts taxonomical affiliation, all PAH-degrading transcripts assigned to Alteromonadales were found enriched at the FL and PA cohorts of the SML, suggesting this group played a relevant role in biodegradation processes in this layer (Supplementary Figure 7). At the SSL PA, the whole battery of PAHs-degrading genes were enriched, being homogenously distributed across phylogenetically distinct taxa (Figure 4 and Supplementary Figure 7). At the SSL FL fraction, the genes from the upper pathway of PAHs degradation were enriched (from the RHD to the prior steps toward catechol or protocatechuate), but later steps (PF00561 and PF07836) toward tricarboxylic acid (TCA) cycle were found depleted. Thus, the metatranscriptomic profiles after 24 h captured different stages of the degradation of PAHs, steered by different taxa, at both layers (SML and SSL) and for both fractions (PA and FL). After 24 h, PA Alteromonadales in the SML had already degraded a significant fraction of HMW PAHs adsorbed to POC, and then, biodegradation was occurring mainly in the SML FL and SSL PA fractions. The more pronounced decrease of HMW PAHs than LMW PAHs is consistent with these different kinetics in bacterial degradation for both fractions.

Importantly, PAHs biodegradation does not occur in a nutshell. It requires other metabolisms involving strategies to chemically sense hydrocarbons, to move toward them, to produce substances modifying their bioavailability, and to deploy an array of cell detoxification strategies to cope with PAHs toxicity (Joye et al., 2016, 2018). Consistently, genes related to chemotaxis and to motility on one side and to stress response regulation and detoxifying multidrug efflux pumps on the other were found enriched in FL SML community exposed to PAHs (*t*-test, *P* < 0.05, Supplementary Figure 8). Similarly, at the PA cohort, a significant increase was observed for transcripts associated with the functional categories of “flagellar biosynthesis protein FliS” and “Widespread colonization island,” the latter category being involved in biofilm formation and surface colonization (Planet et al., 2003; Caruso, 2020) (*t*-test, *P* < 0.05). At SSL, significant changes were observed uniquely for the FL fraction (Supplementary Figure 8).

All together, these results support SML as a hotspot habitat for PAHs degradation in Antarctic coastal waters (Supplementary Figure 9). The surprising fact that fast PAHs degradation is observed for the HMW PAHs at the SML might be related to the synergistic combination of several factors: (1) traditionally, HMW PAHs have been considered to be more persistent than LMW PAHs due to strong sorption of HMW PAHs to black carbon. However, black carbon concentrations are extremely low in Antarctica, and thus, it does not lower the bioavailable concentrations. The higher hydrophobicity of HMW PAHs drives their partitioning to microorganisms and detritus, which are the major organic matter pools, facilitating their biodegradation by particle-associated bacteria; (2) organic pollutants are enriched at the SML (Cincinelli et al., 2005; Stortini et al., 2009; Casas et al., 2020); and (3) there is the enrichment of hydrocarbonoclastic and organic pollutant-degrading bacterial communities in the SML compared with subsurface waters (Coelho et al., 2011; Martinez-Varela et al., 2020). These findings need deeper characterization in further studies, which will elucidate the potential Antarctic ecosystem vulnerability to anthropogenic stressors such as semivolatile organic pollutants.

## Conclusion

Microbial communities from the SML and SSL were exposed to environmental concentrations of PAHs at the coastal Livingston Island (Antarctica) for 24 h. Significantly higher PAHs removal rates were measured at the SML community, particularly for the most hydrophobic PAHs with higher particle sorption tendency. Consistently, the particle-associated microbial community at the SML showed the highest number of significant changes based on 16S rRNA amplicon sequencing. Particularly, the hydrocarbonoclastic *Pseudoalteromonas* significantly increased its relative abundance, becoming a dominant community player in PAHs-exposed communities. Significant differentially expressed genes between PAHs-exposed communities and controls were more abundant at the SML than in SSL metatranscriptomes after 24 h, mostly harbored by Alteromonadales. The pivotal role played by *Pseudoalteromonas* at the SML over other obligate hydrocarbonoclastic bacteria present at initial samples suggests that other physiological capacities like mechanisms of protection against oxidative stress are equally relevant to succeed in a harsh environment such as the SML. All together, these results support SML as a hotspot habitat for PAHs degradation in Antarctic coastal waters that will need deeper characterization in further studies in this endangered pristine ecosystem.

## Data Availability Statement

The datasets presented in this study can be found in online repositories. The names of the repository/repositories and accession number(s) can be found below: https://www.ncbi.nlm.nih.gov/, PRJNA739708 and PRJEB54011; https://www.ncbi.nlm.nih.gov/, SUB9892364.

## Author Contributions

AM-V, MV-C, and JD designed the experimental setup and wrote the manuscript. AM-V and GC conducted the field sampling and the experiment. AM-V and NB analyzed and quantified the PAH concentrations. AM-V, MV-C, and BP performed the molecular work and data analyses. All authors discussed the results and implications and commented on the final version of the manuscript.

## Conflict of Interest

The authors declare that the research was conducted in the absence of any commercial or financial relationships that could be construed as a potential conflict of interest.

## Publisher’s Note

All claims expressed in this article are solely those of the authors and do not necessarily represent those of their affiliated organizations, or those of the publisher, the editors and the reviewers. Any product that may be evaluated in this article, or claim that may be made by its manufacturer, is not guaranteed or endorsed by the publisher.
