# Supplementary material for: Polycyclic Aromatic Hydrocarbon Degradation in the Sea-Surface Microlayer at Coastal Antarctica
Source: Front Microbiol. 2022 Jul 14;13:907265. doi: 10.3389/fmicb.2022.907265 (PMC9329070; doi:10.3389/fmicb.2022.907265)
Supplement: Supplementary file 1 [file Data_Sheet_1.docx]

Submitted to Frontiers in Microbiology

**Polycyclic Aromatic Hydrocarbon degradation in the sea-surface microlayer at coastal Antarctica**

Alícia Martinez-Varela^1^, Gemma Casas^1^, Naiara Berrojalbiz^1^, Benjamin Piña^1^, Jordi Dachs^1^  and Maria Vila-Costa^1^

^1^ Department of Environmental Chemistry, Institute of Environmental Assessment and Water Research, IDAEA-CSIC; Barcelona, Catalunya, Spain

Corresponding author: Maria Vila-Costa

Email: [maria.vila@idaea.csic.es](mailto:maria.vila@idaea.csic.es)

OrcID: 0000-0003-1730-8418

**Contents summary**

**Table S1.** Abiotic and meteorological parameters at initial conditions.

**Table S2.** PAH dissolved phase concentrations time course in each condition, layer and time-point (units are ng L^-1^ and variability is expressed as the standard error of mean).

**Table S3.** Experimental bottles and analyses performed

**Table S4.** Sequencing depths for the 16S rDNA amplicon sequences

**Table S5.** Metatranscriptome library sizes.

**Table S6.** Pfam profiles of proteins involved in PAH degradation

**Table S7**. Subset of genera including previously reported hydrocarbonoclastic bacterial strains and metagenome-assembled genome (MAG) genera retrieved from oil polluted marine environments detected in our experimental dataset.

**Table S8.** Inorganic nutrient concentrations and bacterial abundance measurements for the SML and SSL over the time course of the incubation.

**Table S9.** Community composition of microbial communities at initial time point measured by 16S rRNA gene amplicon sequencing

**Table S10.** Community composition of microbial communities at final time point (24h) measured by 16S rRNA gene amplicon sequencing

**Figure S1**. Map of the sampled sea water.

**Figure S2**. Benchmarked degradation rates

**Figure S3.** Comparison of relative abundance of genes and transcripts between bacterial layers (SML vs SSL) for each bacterial fraction in the rRNA 16S gene pool and the taxonomical assignation of the metatranscriptome at initial conditions.

**Figure S4.** Principal component analysis (PCA) plot showing 16S of rRNA gene at initial conditions from the different sampled communities

**Figure S5.** Relative abundances of hydrocarbonoclastic baterial strains based on the 16S rRNA gene amplicon sequencing. Taxonomical affiliations are based on Table S5.

**Figure S6.** Relative contributions of taxa to the community composition and transcripts pool measured by 16S rRNA gene amplicon sequencing and metatranscriptomics at initial conditions and over the course of the incubation for each experimental condition.

**Figure S7.** Fold-change in expression of genes involved in PAH degradation  between PAH treatments and controls and their taxonomic affiliation.

**Figure S8.**  Fold change of the relative abundance of transcripts within SEED categories between PAH and control treatments

**Figure S9.**  Enrichment factors of the SML PAH degradation rates.

**Table S1.** Abiotic and meteorological parameters at initial conditions in the subsurface layer. a) Ancillary data for the seawater sampling site on 21/02/2018 at 62º38.346' S, 60º23.912' W (Figure S1). b) Meteorological conditions during the sampling event. The values were averaged coinciding the sampling hours (From 8 am to 11.00 am).

| 1. **Ancillary data for the seawater sampling site:** | |
| --- | --- |
| Surface Sea Temperature (˚C) | 1.9 |
| Salinity (PSU) | 33.9 |
| Conductivity | 3.0 |
| Fluorescence (RFU) | 0.5 |
| PAR/Irradiance, Biospherical/Licor | 0.3 |
| Turbidity (FTU) | 3.8 |
| Density | 27.2 |
| **b) Meteorological conditions during the sampling event:** | |
| Wind speed (ms^-1^) | 2.4 |
| Wind direction (°) | 42.5 |
| Temperature (°C) | 3.5 |
| Minimum temp. (°C) | 3.5 |
| Maximum temp. (°C) | 3.6 |
| Relative humidity (%) | 95.1 |
| Precipitation (mm) | 0.0 |
| Atmospheric pressure (hPa) | 993.0 |
| Radiation (Wm^-2^) | 5.7 |

**Table S2.** PAH dissolved phase concentrations time course in each condition, layer and time-point (units are ng L^-1^ and variability is expressed as the standard error of mean).

|  | ABIOTIC CONTROL | |
| --- | --- | --- |
|  | T0 | T24 |
| Fluorene | 20.43 ± 2.55 | 34.25 ± 1.77 |
| Anthracene | 24.48 ± 3.22 | 40.68 ± 2.18 |
| Phenanthrene | 29.98 ± 3.53 | 41.39 ± 1.71 |
| Pyrene | 41.91 ± 3.88 | 53.12 ± 1.43 |
| Fluoranthene | 47.93 ± 4.9 | 59.92 ± 1.78 |
| Crysene | 7.42 ± 0.13 | 9.38 ± 1.74 |
| Benzo(a)anthracene | 23.05 ± 0.32 | 29.07 ± 3.63 |
| Benzo(b)fluoranthene | 41.82 ± 0.33 | 59.85 ± 7.94 |
| Benzo(k)fluoranthene | 24.68 ± 0.37 | 22.68 ± 7.25 |
| Benzo(a)pyrene | 59.55 ± 1.23 | 58.89 ± 14.62 |
| Dibenzo(a,h)anthracene | 35.42 ± 0.03 | 20.26 ± 14.5 |
| Indeno(1,2,3-cd)pyrene | 48.11 ± 0.27 | 34.45 ± 15.62 |
| Benzo(ghi)perylene | 27.38 ± 0.03 | 17 ± 10.5 |
| Σ  LMW PAHs | 74.9 ± 9.29 | 116.32 ± 5.66 |
| Σ  HMW PAHs | 357.27 ± 7.47 | 364.61 ± 79.01 |
| Σ total PAHs | 432.17 ± 16.76 | 480.93 ± 84.68 |

|  | SML CONTROL | | SML PAH TREATMENT | | SSL CONTROL | | SSL PAH TREATMENT | |
| --- | --- | --- | --- | --- | --- | --- | --- | --- |
|  | T0 | T24 | T0 | T24 | T0 | T24 | T0 | T24 |
| Fluorene | 0.12 ± 0.02 | 0.34 ± 0.1 | 13.65 ± 0.32 | 12.92 ± 1.76 | 0.88 ± 0.03 | 0.07 ± 0.07 | 10.73 ± 1.95 | 5.97 ± 0.75 |
| Anthracene | 0.02 ± 0.01 | 0.04 ± 0 | 15.07 ± 2.36 | 9.13 ± 1.49 | 0.11 ± 0.01 | 0.02 ± 0 | 6.52 ± 0.98 | 3.88 ± 1.59 |
| Phenanthrene | 0.27 ± 0.09 | 0.83 ± 0.09 | 17.7 ± 1.42 | 13.92 ± 0.89 | 2.26 ± 0.12 | 0.38 ± 0.06 | 12.34 ± 2.91 | 7.26 ± 1.52 |
| Pyrene | 0.03 ± 0 | 0.05 ± 0.02 | 18.85 ± 3.12 | 11.95 ± 0.32 | 0.1 ± 0 | 0.06 ± 0.01 | 12.23 ± 5.39 | 5.15 ± 1.71 |
| Fluoranthene | 0.03 ± 0 | 0.04 ± 0 | 22.5 ± 3.84 | 13.84 ± 0.37 | 0.09 ± 0 | 0.04 ± 0 | 13.65 ± 5.98 | 6.4 ± 2.15 |
| Crysene | 0.03 ± 0.01 | 0 ± 0 | 15.16 ± 5.03 | 5.56 ± 1.49 | 0.01 ± 0.01 | 0 ± 0 | 1.87 ± 0.08 | 1.98 ± 0.97 |
| Benzo(a)anthracene | 0 ± 0 | 0 ± 0 | 28.63 ± 7.84 | 12.88 ± 2.72 | 0 ± 0 | 0 ± 0 | 5.92 ± 0.02 | 5.52 ± 2.42 |
| Benzo(b)fluoranthene | 0 ± 0 | 0 ± 0 | 70.53 ± 26.91 | 26.73 ± 7.64 | 0 ± 0 | 0 ± 0 | 11.6 ± 0.28 | 10.76 ± 3.58 |
| Benzo(k)fluoranthene | 0 ± 0 | 0 ± 0 | 61.58 ± 24.34 | 21.63 ± 7.79 | 0 ± 0 | 0 ± 0 | 6.52 ± 0.34 | 6.63 ± 2.46 |
| Benzo(a)pyrene | 0 ± 0 | 0 ± 0 | 113.61 ± 36.57 | 46.59 ± 14.13 | 0 ± 0 | 0 ± 0 | 18.09 ± 0.18 | 17.28 ± 6.55 |
| Dibenzo(a,h)anthracene | 0 ± 0 | 0 ± 0 | 106.09 ± 41.28 | 37.15 ± 17.69 | 0 ± 0 | 0 ± 0 | 8.29 ± 1.08 | 7.8 ± 2.81 |
| Indeno  (1,2,3-cd)pyrene | 0.14 ± 0.01 | 0.03 ± 0.03 | 118.19 ± 46 | 44.31 ± 17.5 | 0.11 ± 0.02 | 0.02 ± 0.02 | 13.05 ± 0.01 | 12.89 ± 4.6 |
| Benzo(ghi)perylene | 0.09 ± 0 | 0.02 ± 0.02 | 78.92 ± 33.68 | 28.63 ± 12.77 | 0.07 ± 0 | 0.02 ± 0.02 | 6.84 ± 0.42 | 6.51 ± 2.08 |
| Σ  LMW PAHs | 0.41 ± 0.11 | 1.21 ± 0.19 | 46.42 ± 4.1 | 35.96 ± 4.14 | 3.25 ± 0.16 | 0.47 ± 0.01 | 29.59 ± 5.84 | 17.11 ± 3.87 |
| Σ  HMW PAHs | 0.32 ± 0.03 | 0.14 ± 0.06 | 634.06 ± 228.61 | 249.27 ± 82.41 | 0.38 ± 0.03 | 0.13 ± 0.02 | 98.06 ± 12.55 | 80.91 ± 29.33 |
| Σ total PAHs | 0.74 ± 0.14 | 1.36 ± 0.26 | 680.48 ± 232.7 | 285.23 ± 86.55 | 3.63 ± 0.19 | 0.6 ± 0.03 | 127.65 ± 18.38 | 98.02 ± 33.2 |

**Table S3.** Experimental conditions and analyses performed.

| **Bottle** | **Time point** | **Layer/**  **water** | **Treatment** | **Replicate** | **PAH concentrations** | **16S rRNA** | **MetaT** |
| --- | --- | --- | --- | --- | --- | --- | --- |
| BOT1 | T0 | SML | PAH | A | X | X | X |
| BOT2 | T0 | SML | PAH | B | X | X | X |
| BOT3 | T0 | SSL | PAH | A | X | X | X |
| BOT4 | T0 | SSL | PAH | B | X | X | X |
| BOT5 | T0 | SML | control | A | X | X | X |
| BOT6 | T0 | SML | control | B | X | X | X |
| BOT7 | T0 | SSL | control | A | X | X | X |
| BOT8 | T0 | SSL | control | B | X | X | X |
| BOT9 | T24 | SML | PAH | A | X | X | X |
| BOT10 | T24 | SML | PAH | B | X | X | X |
| BOT11 | T24 | SSL | PAH | A | X | X | X |
| BOT12 | T24 | SSL | PAH | B | X | X | X |
| BOT13 | T24 | SML | control | A | X | X | X |
| BOT14 | T24 | SML | control | B | X | X | X |
| BOT15 | T24 | SSL | control | A | X | X | X |
| BOT16 | T24 | SSL | control | B | X | X | X |
| BOT17 | T0 | HPLC | PAH | A | X |  |  |
| BOT18 | T0 | HPLC | PAH | B | X |  |  |
| BOT19 | T24 | HPLC | PAH | A | X |  |  |
| BOT20 | T24 | HPLC | PAH | B | X |  |  |

**Table S4.** Sequencing depths for the 16S rDNA amplicon sequences (SML: sea surface microlayer; SSL: subsurface layer; FL: Free Living, PA: Particle-Associated)

| **Sample ID**  **SRA** | **Seq. depth** | **Unique ASV** | **Cohort** | **Time point** | **Layer** | **Treatment** | **Replicate** |
| --- | --- | --- | --- | --- | --- | --- | --- |
| U1 | 137739 | 237 | FL | T0 | SML | PAH | A |
| U10 | 53255 | 155 | FL | T24 | SML | PAH | B |
| U11 | 19174 | 137 | FL | T24 | SSL | PAH | A |
| U12 | 17240 | 127 | FL | T24 | SSL | PAH | B |
| U13 | 37126 | 143 | FL | T24 | SML | control | A |
| U14 | 51696 | 154 | FL | T24 | SML | control | B |
| U15 | 73586 | 220 | FL | T24 | SSL | control | A |
| U16 | 87079 | 202 | FL | T24 | SSL | control | B |
| U3 | 61661 | 190 | FL | T0 | SSL | PAH | A |
| U33 | 40456 | 161 | PA | T0 | SML | PAH | A |
| U35 | 46465 | 196 | PA | T0 | SSL | PAH | A |
| U37 | 29167 | 154 | PA | T0 | SML | control | A |
| U39 | 33197 | 93 | PA | T0 | SSL | control | A |
| U41 | 28337 | 158 | PA | T24 | SML | PAH | A |
| U42 | 25367 | 132 | PA | T24 | SML | PAH | B |
| U43 | 22141 | 83 | PA | T24 | SSL | PAH | A |
| U44 | 11664 | 68 | PA | T24 | SSL | PAH | B |
| U45 | 30736 | 156 | PA | T24 | SML | control | A |
| U46 | 40818 | 127 | PA | T24 | SSL | control | A |
| U47 | 21916 | 87 | PA | T24 | SSL | control | B |
| U5 | 44108 | 159 | FL | T0 | SML | control | A |
| U7 | 72715 | 179 | FL | T0 | SSL | control | A |
| U9 | 19202 | 72 | FL | T24 | SML | PAH | A |

**Table S5.** Metatranscriptome library sizes (SML: sea surface microlayer; SSL: subsurface layer; FL: Free Living, PA: Particle-Associated)

| **Sample ID**  **SRA** | **Library size** | **Cohort** | **Time point** | **Layer** | **Treatment** | **Replicate** |
| --- | --- | --- | --- | --- | --- | --- |
| S1_126UDI | 21222581 | PA | T24 | SML | control | A |
| S1_138UDI | 4567076 | PA | T24 | SSL | control | A |
| S2_115UDI | 8280899 | FL | T0 | SML | control | A |
| S2_139UDI | 8111315 | PA | T24 | SML | PAH | A |
| S2_19UDI | 5331503 | FL | T0 | SSL | control | A |
| S2_31UDI | 4484565 | FL | T24 | SML | control | A |
| S2_43UDI | 5784411 | PA | T0 | SML | control | A |
| S2_7UDI | 3646155 | PA | T24 | SSL | control | B |
| S3_100UDI | 6269886 | FL | T0 | SML | control | B |
| S3_112UDI | 10768454 | FL | T0 | SSL | control | B |
| S3_124UDI | 5849038 | FL | T24 | SML | PAH | A |
| S3_136UDI | 6173900 | FL | T24 | SML | PAH | B |
| S3_16UDI | 7988369 | FL | T0 | SML | PAH | B |
| S3_17UDI | 6779729 | FL | T24 | SSL | PAH | B |
| S3_28UDI | 5611156 | FL | T0 | SSL | PAH | A |
| S3_40UDI | 10205518 | FL | T0 | SSL | PAH | B |
| S3_4UDI | 5294704 | FL | T0 | SML | PAH | A |
| S3_5UDI | 9107911 | FL | T24 | SSL | PAH | A |
| S4_101UDI | 9715689 | FL | T24 | SSL | control | B |
| S4_102UDI | 5138788 | PA | T24 | SSL | PAH | A |
| S4_113UDI | 5716160 | PA | T0 | SML | PAH | A |
| S4_114UDI | 3082398 | PA | T24 | SSL | PAH | B |
| S4_125UDI | 27028349 | PA | T0 | SML | PAH | B |
| S4_137UDI | 7594777 | PA | T0 | SSL | PAH | A |
| S4_18UDI | 6513608 | PA | T0 | SSL | control | A |
| S4_30UDI | 7222310 | PA | T0 | SSL | control | B |
| S4_41UDI | 7748495 | FL | T24 | SSL | control | A |
| S4_42UDI | 6187946 | PA | T24 | SML | PAH | B |
| S4_6UDI | 7096732 | PA | T0 | SML | control | B |
| S7_103UDI | 2970296 | PA | T24 | SML | control | B |
| S7_31UDI | 2825865 | FL | T24 | SML | control | B |

**Table S6.** Pfam profiles of proteins involved in PAH degradation**.** In bold, the initiating PAH degrading enzyme, catalyzing the hydroxylation of the aromatic ring. (Pfam: The protein families database number; EC: Enzyme Comission number)

| Pfam | Pathway | Name | gene | E.C. no. |
| --- | --- | --- | --- | --- |
| **PF00848** | Upstream PAH degradation | **Ring-hydroxylating dioxygenase** | **nahA** | **1.14.12.12** |
| PF00171 |  | Salicylaldehyde dehydrogenase | nahF | 1.2.1.65 |
| PF00596 |  | 3,4-dihydroxyphthalate decarboxylase | padC | 4.1.1.69 |
| PF00701 |  | Dihydrodipicolinate synthetase | nahE | 4.1.2.45 |
| PF00775 |  | Protocatechuate 3,4-dioxygenase beta chain | pcaH | 1.13.11.3 |
| PF00106 |  | 1,6-dihydroxycyclohexa-2,4-diene-1-carboxylate dehydrogenase | benD | 1.3.1.25 |
| PF00903 |  | Glyoxalase dioxygenase | nahC | 1.13.11.56 |
| PF01323 |  | 2-hydroxychromene-2-carboxylate isomerase | nahD | 5.99.1.4 |
| PF01494 |  | Salicylate hydroxylase | nahG | 1.14.13.1 |
| PF09084 |  | 4,5-dihydroxyphthalate decarboxylase | pht5 | 4.1.1.55 |
| PF00561 | Catechol degradation | 2-hydroxymuconate semialdehyde hydrolase | xylF | 3.7.1.9 |
| PF00903 |  | Metapyrocatechase | xylE | 1.13.11.2 |
| PF02746 |  | Muconate cycloisomerase 1 | catB | 5.5.1.1 |
| PF04444 |  | Catechol 1,2-dioxygenase | catA | 1.13.11.1 |
| PF07836 |  | 4-hydroxy-2-oxovalerate aldolase | xylK | 4.1.3.39 |
| PF02426 |  | Muconolactone Delta-isomerase | catC | 5.3.3.4 |
| PF00206 | Benzoate and protocatechuate degradation | 3-carboxy-cis,cis-muconate cycloisomerase | pcaB | 5.5.1.2 |
| PF00561 |  | 3-oxoadipate enol-lactonase | pcaD | 3.1.1.24 |
| PF01144 |  | Coenzyme A transferase | pcaI | 2.8.3.6 |
| PF02627 |  | 4-carboxymuconolactone decarboxylase | pcaC | 4.1.1.44 |

**Table S7**. Subset of genera including previously reported hydrocarbonoclastic bacterial strains and metagenome-assembled genome (MAG) genera retrieved from oil polluted marine environments(Lozada *et al.* 2014; Karthikeyan *et al.* 2020) and detected in our experimental dataset. As some of these taxa have also been reported to exudate biosurfactants or form biofilms, the type and citation is also given. In bold, marine biosurfactant-producing bacterial strains within the genera (HC : Hydrocarbonoclastic).

| **Label** | **Phylum/Class** | **Genus** | **Biosurfactant-producing bacteria** | |
| --- | --- | --- | --- | --- |
| HC Actinobacteria | Phylum: Actinobacteria | *Arthrobacter* | **Glycolipid** | (Schulz *et al.* 1991) |
|  |  | *Nocardioides* | Glycolipid | (Vasileva-Tonkova and Gesheva 2005) |
| HC Alpha-proteobacteria | Phylum: Proteobacteria  Class: Alpha-proteobacteria | *Sulfitobacter* |  |  |
|  |  | *Sphingomonas* | Polymeric surfactant | (Santos *et al.* 2016) |
|  |  | *Roseobacter* | Biofilm formation (high temperature adaptation) | (Kent *et al.* 2018) |
| HC Bacteroidia | Phylum: Bacteroidetes  Class: Flavobacteriia | *Flavobacterium* | Biosurfactant  (From contaminated arid soils) | (Bodour *et al.* 2003) |
| HC Gamma-proteobacteria | Phylum: Proteobacteria  Class: Gamma-proteobacteria | *Acinetobacter* | **Polymeric biosurfactant/**  **bioemulsifier** | (Rosenberg *et al.* 1988) |
|  |  | Arenimonas |  |  |
|  |  | *Colwellia* | **Biosurfactant** | (Mapelli *et al.* 2017) |
|  |  | *Glaciecola* | **Biosurfactant/ bioemulsifier** | (Dang *et al.* 2016) |
|  |  | *Halomonas* | **Polymeric biosurfactant/**  **bioemulsifier, glycolipid and Exopolysacharides**  (Isolated from Surface waters affected by Deep Horizon Oil spill in the Gulf of Mexico) | (Gutiérrez *et al.* 2007; Gutierrez *et al.* 2013) |
|  |  | *Marinomonas* | **Biosurfactant** | (Dang *et al.* 2016) |
|  |  | Oleiphilus |  |  |
|  |  | *Oleispira* |  |  |
|  |  | *Pseudoalteromonas* | **Exopolysacharides (complex Surface active polymer )** | (Malavenda *et al.* 2015; Roca *et al.* 2016) |
|  |  | *Pseudomonas* | **Polymeric biosurfactant/**  **bioemulsifier, lipopeptides, glycolipids and particulate surfactant** | (Wittgens *et al.* 2017; Domingues *et al.* 2020) |
|  |  | Pshychrobacter | **Biosurfactant** | (Domingues *et al.* 2020; Trudgeon *et al.* 2020) |
|  |  | *Shewanella* | **Biosurfactant** | (Antoniou *et al.* 2015) |
|  |  | *Vibrio* | **Exopolysacharides (complex Surface active polymer )** | (Satpute *et al.* 2010) |
|  |  | *Woeseia* |  |  |
| All HCB |  | *Includes all listed above |  |  |

**Table S8.** Inorganic nutrient concentrations and bacterial abundance measurements for the SML and SSL over the time course of the incubation. Variability is expressed with standard deviation. (SML: sea surface microlayer; SSL: subsurface layer; LNA: Low Nucleic Acid; HNA: High Nucleic Acid; All BA : All Bacterial Abundance; FL: Free living, PA: Particle-associated)

|  |  | **T0** | | **T24** | | | |
| --- | --- | --- | --- | --- | --- | --- | --- |
|  |  | **SML** | **SSL** | **SML** | | **SSL** | |
|  |  |  |  | **Control** | **PAH** | **Control** | **PAH** |
| **N-NH_4_^+^ (μmolL^-1^)** | | 4.9 ± 5.3 | 4.3 ± 2.6 | 5.1 ± 3.3 | 2.9 ± 0.3 | 9.2 ± 7.1 | 2.5 ± 0.6 |
| **NO_−3_ + NO_-2_ (μmol L^-1^)** | | 23.2 ± 2.6 | 18 ± 6.3 | 22.6 ± 4 | 24 ± 0.4 | 16.6 ± 6.5 | 23.1 ± 1.5 |
| **-PO_4_^3-^**  **(μmol L^-1^)** | | 1.6 ± 0.2 | 1.4 ± 0.2 | 1.4 ± 0.1 | 1.4 ± 0 | 1.3 ± 0.2 | 1.5 ± 0.1 |
| **LNA**  **(Cells mL^-1^)** | | 131408.1± 60065.5 | 157930.8 ± 41564.9 | 165009.0±  19286.8 | 153743±  64633.4 | 132735.1±  34322.9 | 163931.7±  19980.9 |
| **HNA**  **(Cells mL^-1^)** | | 428419.8 ± 187039.5 | 498016.0 ± 157063.5 | 588590.6±  107145.4 | 338282 ±  242176.2 | 446875.6±  138310.3 | 474584.1±  93771 |
| **All BA**  **(Cells mL^-1^)** | | 559827.9 ± 245034.8 | 655946.8 ± 195410.2 | 753599.7±  126070.3 | 492025±  239429.3 | 579610.7±  172019 | 638515.7±  112373 |
| **Bacterial abundance yields**  **(Cells ml^-1^ h^-1^)** | |  |  | 0.01±0.06 | 0.00±0.03 | -0.002±0.008 | -0.004±0.01 |

**Table S9.** Community composition of microbial communities at initial time point measured by 16S rRNA gene amplicon sequencing (Relative abundances in % of total reads and variability expressed in standard deviation, SML: sea surface microlayer; SSL: subsurface layer; FL: Free Living, PA: Particle Associated; HC : Hydrocarbonoclastic). Notice that counts of general taxonomical groups include HC bacteria.

|  | SML | | SSL | |
| --- | --- | --- | --- | --- |
|  | FL | PA | FL | PA |
| Actinobacteriota | 0.05 ± 0.04 | 0.29 ± 0.37 | 0.03 ± 0 | 0.56 ± 0.38 |
| Alphaproteobacteria | 21.74 ± 1.13 | 15.06 ± 2.28 | 21.3 ± 0.46 | 19.36 ± 4.75 |
| SAR11 clade | 8 ± 0.63 | 2.64 ± 0.1 | 6.99 ± 0.1 | 2.36 ± 0.35 |
| Caulobacterales | 0 | 0.05 ± 0.03 | 0 | 0.13 ± 0 |
| Rhodobacterales | 11.91 ± 0.51 | 6.62 ± 0.58 | 12.74 ± 0.61 | 7.87 ± 0.14 |
| Rhodospirillales | 0.55 ± 0.01 | 0.45 ± 0.16 | 0.56 ± 0.07 | 0.54 ± 0.08 |
| Sphingomonadales | 0 | 0.05 ± 0 | 0 | 0.11 ± 0.03 |
| Rickettsiales | 0.38 ± 0.04 | 4.94 ± 1.82 | 0.23 ± 0.1 | 8.15 ± 5.42 |
| Gammaproteobacteria | 36.04 ± 0.29 | 33.39 ± 4.62 | 34.66 ± 0.67 | 26.42 ± 2.77 |
| Alteromonadales | 1.97 ± 1.66 | 7.2 ± 4.99 | 0.15 ± 0.06 | 8.09 ± 1.71 |
| Burkholderiales | 0.68 ± 0.06 | 0.14 ± 0 | 0.71 ± 0.25 | 0.13 ± 0.05 |
| Cellvibrionales | 6.61 ± 0.19 | 3.62 ± 1.05 | 7.45 ± 0.34 | 3.05 ± 1.19 |
| Oceanospirillales | 19.08 ± 0.83 | 12.6 ± 0.63 | 20.11 ± 0.69 | 11.73 ± 2.25 |
| Pseudomonadales | 1.02 ± 0.17 | 7.51 ± 2.61 | 0.09 ± 0.04 | 0.16 ± 0 |
| SAR86 clade | 2.54 ± 0.2 | 0.63 ± 0.15 | 2.1 ± 0.47 | 0.44 ± 0.22 |
| Thiotrichales | 0.27 ± 0.02 | 0.49 ± 0 | 0.21 ± 0.02 | 0.37 ± 0.02 |
| Other gammaproteobacteria | 3.87 ± 0.28 | 1.43 ± 0.06 | 3.85 ± 0.09 | 2.53 ± 0.36 |
| Bacteroidota | 41.8 ± 0.91 | 50.22 ± 6.71 | 43.66 ± 0.27 | 52.88 ± 6.71 |
| Cytophagales | 0.87 ± 0.02 | 4.19 ± 1.3 | 0.86 ± 0.08 | 4.94 ± 0.47 |
| Flavobacteriales | 40.92 ± 0.91 | 45.52 ± 5.42 | 42.8 ± 0.36 | 47.35 ± 7.03 |
| Verrucomicrobiota | 0.08 ± 0.04 | 0.23 ± 0.02 | 0.08 ± 0.01 | 0.26 ± 0 |
| Other Bacterial groups | 0.05 ± 0 | 0.33 ± 0.03 | 0.08 ± 0.03 | 0.54 ± 0.08 |
|  |  |  |  |  |
| All HCB | 7.72 ± 1.92 | 18.18 ± 2.1 | 5.95 ± 0.55 | 12.74 ± 0.69 |
| HC Actinobacteria | 0 | 0.04 ± 0 | 0.02 ± 0 | 0.56 ± 0.38 |
| HC Bacteroidia | 0.03 ± 0 | 0.16 ± 0.02 | 0.04 ± 0 | 0.3 ± 0 |
| HC Alphaproteobacteria | 4.74 ± 0.08 | 3.38 ± 0.45 | 5.69 ± 0.57 | 3.9 ± 0.5 |
| *Sulfitobacter* | 4.74 ± 0.08 | 3.36 ± 0.43 | 5.69 ± 0.57 | 3.9 ± 0.5 |
| HC Gammaproteobacteria | 2.96 ± 1.82 | 14.62 ± 2.5 | 0.22 ± 0.04 | 8.14 ± 1.78 |
| *Colwellia* | 0.03 ± 0 | 0.05 ± 0 | 0.1 ± 0.04 | 7.62 ± 1.83 |
| *Pseudoalteromonas* | 1.91 ± 1.61 | 6.92 ± 5.28 | 0.06 ± 0 | 0.11 ± 0.11 |
| *Psychrobacter* | 1.02 ± 0.17 | 7.47 ± 2.56 | 0.09 ± 0.04 | 0.16 ± 0 |

**Table S10.** Community composition of microbial communities at final time point (24 h) measured by 16S rRNA gene amplicon sequencing. (Relative abundances in % of total reads and variability expressed in standard deviation, SML: sea surface microlayer; SSL: subsurface layer; FL: Free Living, PA: Particle-Associated; HC : Hydrocarbonoclastic). Notice that counts of general taxonomical groups include HC bacteria.

|  | SML | | | | SSL | | | |
| --- | --- | --- | --- | --- | --- | --- | --- | --- |
|  | FL | | PA | | FL | | PA | |
|  | CNTRL | PAH | CNTRL | PAH | CNTRL | PAH | CNTRL | PAH |
| Actinobacteriota | 0.03 ± 0.01 | 0.01 ± 0 | 0.11 | 0.05 ± 0 | 0.05 ± 0.01 | 0.05 ± 0.02 | 0.7 ± 0.41 | 0.68 ± 0 |
| Alphaproteobacteria | 26.02 ± 1.18 | 25.34 ± 0.28 | 14.17 | 12.11 ± 0.22 | 24.5 ± 0.37 | 25.38 ± 1.2 | 19.77 ± 0.23 | 20.63 ± 1.55 |
| SAR11 clade | 7.64 ± 0.32 | 7.34 ± 1.39 | 1.64 | 2 ± 0.5 | 7.64 ± 0.12 | 7.33 ± 0.28 | 1.62 ± 0.65 | 1.85 ± 0.15 |
| Rhodobacterales | 16.6 ± 0.52 | 16.48 ± 1.08 | 7.62 | 5.39 ± 0.67 | 15.1 ± 0.47 | 15.95 ± 0.9 | 8.87 ± 1.32 | 8.39 ± 0.21 |
| Rhodospirillales | 0.5 ± 0.01 | 0.44 ± 0.04 | 0.35 | 0.27 ± 0.07 | 0.41 ± 0.01 | 0.57 ± 0.04 | 0.17 ± 0 | 0.54 ± 0.08 |
| Rickettsiales | 0.6 ± 0.27 | 0.26 ± 0.04 | 4.17 | 4.13 ± 0.94 | 0.56 ± 0.07 | 0.63 ± 0 | 8.49 ± 1.91 | 9.02 ± 2.06 |
| Gammaproteobacteria | 36.85 ± 0.38 | 39.27 ± 0.26 | 43.95 | 53.09 ± 0.37 | 35.56 ± 0.64 | 34.94 ± 0.4 | 29.01 ± 3.37 | 28.43 ± 1.12 |
| Alteromonadales | 0.84 ± 0.29 | 5.86 ± 0.96 | 4.26 | 26.39 ± 0.55 | 0.21 ± 0.01 | 0.22 ± 0.06 | 13.31 ± 3.97 | 15.44 ± 0.8 |
| Burkholderiales | 0.63 ± 0.11 | 0.76 ± 0.08 | 0 | 0.18 ± 0 | 0.72 ± 0.06 | 0.7 ± 0.08 | 0 | 0.1 ± 0 |
| Cellvibrionales | 7.78 ± 0.08 | 7.59 ± 1.73 | 4.17 | 2.8 ± 0.28 | 7.63 ± 0.21 | 7.58 ± 0.17 | 2.24 ± 0.24 | 1.77 ± 0.99 |
| Oceanospirillales | 20.65 ± 0.15 | 17.87 ± 2.39 | 13.01 | 7.39 ± 0.24 | 19.97 ± 0.57 | 19.69 ± 0.38 | 9.82 ± 0.38 | 8.24 ± 1.09 |
| Pseudomonadales | 1.26 ± 1.31 | 1.12 ± 0.08 | 20.71 | 15.05 ± 0.88 | 0.07 ± 0.01 | 0.04 ± 0 | 0.17 ± 0 | 0.29 ± 0 |
| SAR86 clade | 2.39 ± 0.52 | 2.44 ± 0.15 | 0.13 | 0.36 ± 0 | 2.87 ± 0.08 | 2.48 ± 0.32 | 0.66 ± 0 | 0.45 ± 0.09 |
| Thiotrichales | 0.13 ± 0.02 | 0.1 ± 0.07 | 0.3 | 0.41 ± 0.2 | 0.14 ± 0.05 | 0.18 ± 0.01 | 0.7 ± 0.06 | 0.23 ± 0.09 |
| Other gammaproteobacteria | 3.19 ± 0.96 | 3.52 ± 0.26 | 1.37 | 0.78 ± 0.07 | 3.96 ± 0.17 | 4.07 ± 0.15 | 2.53 ± 0.87 | 2.12 ± 0.12 |
| Other proteobacteria | 0.14 ± 0.04 | 0.1 ± 0.04 | 0.43 | 0.15 ± 0.03 | 0.21 ± 0.02 | 0.13 ± 0.04 | 0.17 ± 0 | 0 |
| Bacteroidota | 36.77 ± 0.89 | 35.17 ± 0.66 | 40.59 | 34.16 ± 0.47 | 39.33 ± 0.96 | 39.19 ± 1.72 | 49.48 ± 2.43 | 49.51 ± 0.88 |
| Cytophagales | 0.75 ± 0.05 | 0.49 ± 0.22 | 3.04 | 2.54 ± 0.17 | 1.02 ± 0.09 | 0.89 ± 0.15 | 5.06 ± 0.37 | 4.28 ± 0.56 |
| Flavobacteriales | 36 ± 0.91 | 34.65 ± 0.91 | 37.19 | 31.08 ± 0.99 | 38.28 ± 0.85 | 38.3 ± 1.56 | 43.64 ± 2.39 | 44.5 ± 0.45 |
| Other Bacterial groups | 0.03 ± 0.01 | 0.03 ± 0 | 0.3 | 0.1 ± 0.08 | 0.14 ± 0.09 | 0.11 ± 0.01 | 0.79 ± 0.41 | 0.79 ± 0.39 |
|  |  |  |  |  |  |  |  |  |
| All HCB | 10.97 ± 0.54 | 15.97 ± 0.12 | 29.81 | 45.33 ± 1 | 8.68 ± 0.14 | 9.05 ± 0.11 | 18.9 ± 3.69 | 21.27 ± 0.52 |
| HC Actinobacteria | 0 | 0 | 0 | 0 | 0.03 ± 0.01 | 0.05 ± 0.02 | 0.7 ± 0.41 | 0.68 ± 0 |
| HC Alphaproteobacteria | 8.83 ± 0.53 | 9.02 ± 0.88 | 4.42 | 3.55 ± 0.26 | 8.4 ± 0.13 | 8.79 ± 0.06 | 4.85 ± 1.01 | 4.96 ± 1.52 |
| *Sphingomonas* | 0 | 0 | 0 | 0.02 ± 0 | 0 | 0 | 0 | 0 |
| *Sulfitobacter* | 8.83 ± 0.53 | 9.02 ± 0.88 | 4.42 | 3.53 ± 0.25 | 8.4 ± 0.13 | 8.79 ± 0.06 | 4.85 ± 1.01 | 4.96 ± 1.52 |
| HC Gammaproteobacteria | 2.13 ± 1.06 | 6.95 ± 1.01 | 25.15 | 41.54 ± 0.54 | 0.25 ± 0.02 | 0.21 ± 0.14 | 13.35 ± 3.09 | 15.97 ± 1.56 |
| *Colwellia* | 0.02 ± 0.02 | 0.03 ± 0 | 0.3 | 0.61 ± 0.24 | 0.17 ± 0.01 | 0.11 ± 0.01 | 12.69 ± 4.02 | 14.98 ± 0.76 |
| *Marinomonas* | 0.06 ± 0 | 0.02 ± 0 | 0.43 | 0.22 ± 0 | 0 | 0 | 0 | 0 |
| *Oleispira* | 0 | 0 | 0 | 0 | 0.01 ± 0 | 0 | 0.58 ± 0 | 0.78 ± 0 |
| *Pseudoalteromonas* | 0.82 ± 0.27 | 5.81 ± 0.9 | 3.72 | 25.67 ± 0.28 | 0.01 ± 0 | 0.14 ± 0 | 0 | 0 |
| *Psychrobacter* | 1.24 ± 1.29 | 1.12 ± 0.08 | 18.29 | 15.05 ± 0.88 | 0.07 ± 0.01 | 0.04 ± 0 | 0.17 ± 0 | 0.29 ± 0 |
| HC Bacteroidia | 0.02 ± 0 | 0 | 0.19 | 0.25 ± 0.2 | 0.01 ± 0 | 0 | 0 | 0 |

**Figure S1**. Map of the sampled sea water (sea surface microlayer and subsurface water) for the incubation experiment. Coordinates: 62º38.346' S, 60º23.912'W.


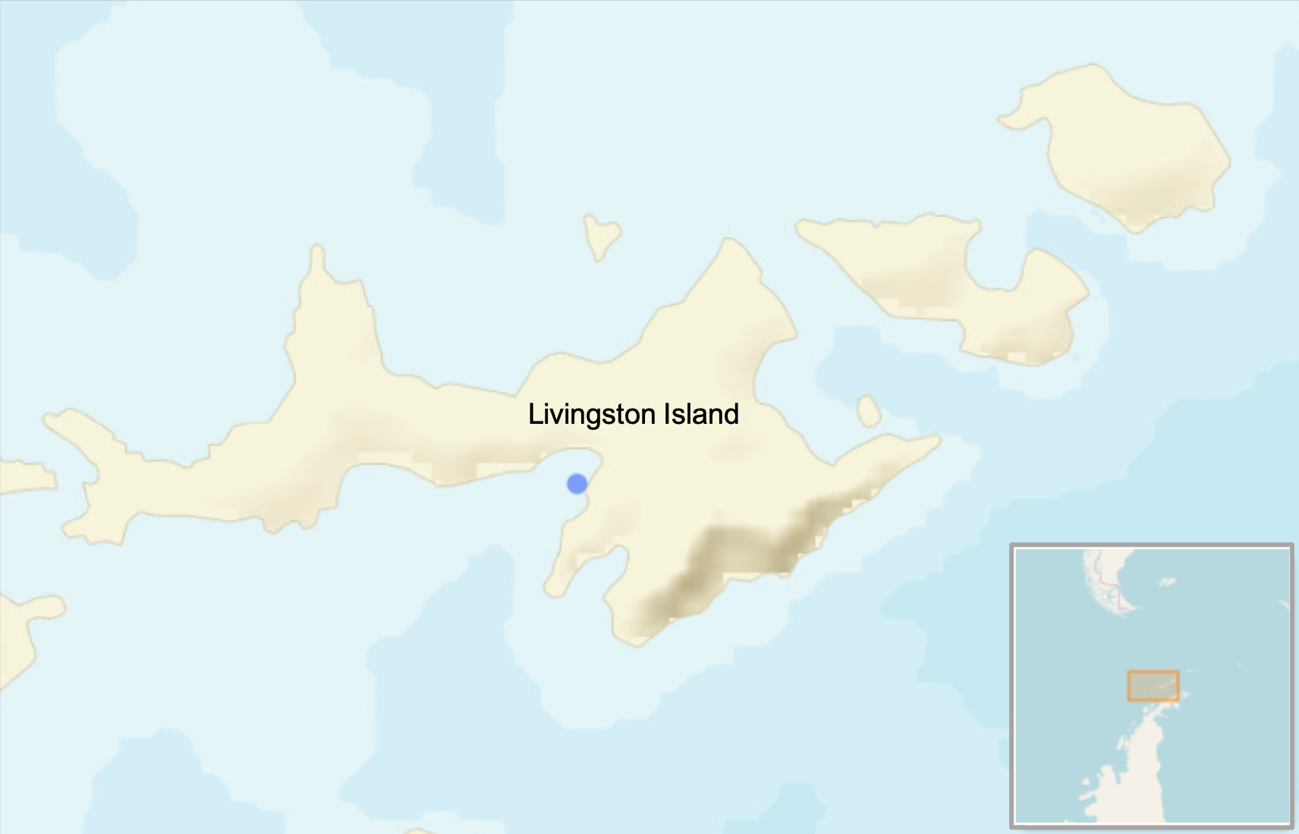


**Figure S2.** PAH degradation rates in the sea surface microlayer (SML) and the subsurface layer (SSL) for treatments and controls using concentrations corrected by benchmarking approach using phenanthrene (PHE). Significant differences between layers (Mann-Whitney test, p<0.05) are labeled with an asterisk at the SML panel only.

**Figure S3.** Comparison of relative abundance of genes and transcripts between bacterial layers (SML vs SSL) for each bacterial fraction in the rRNA 16S gene pool and the taxonomical assignation of the metatranscriptome at initial conditions. Significant statistical differences in the relative abundances between layers were detected by t-test (labeled with an *). (SML: sea surface microlayer; SSL: subsurface layer)

**Figure S4.** Principal component analysis (PCA) plot showing 16S of rRNA gene at initial conditions from the different sampled communities. (SML: sea surface microlayer; SSL: subsurface layer)


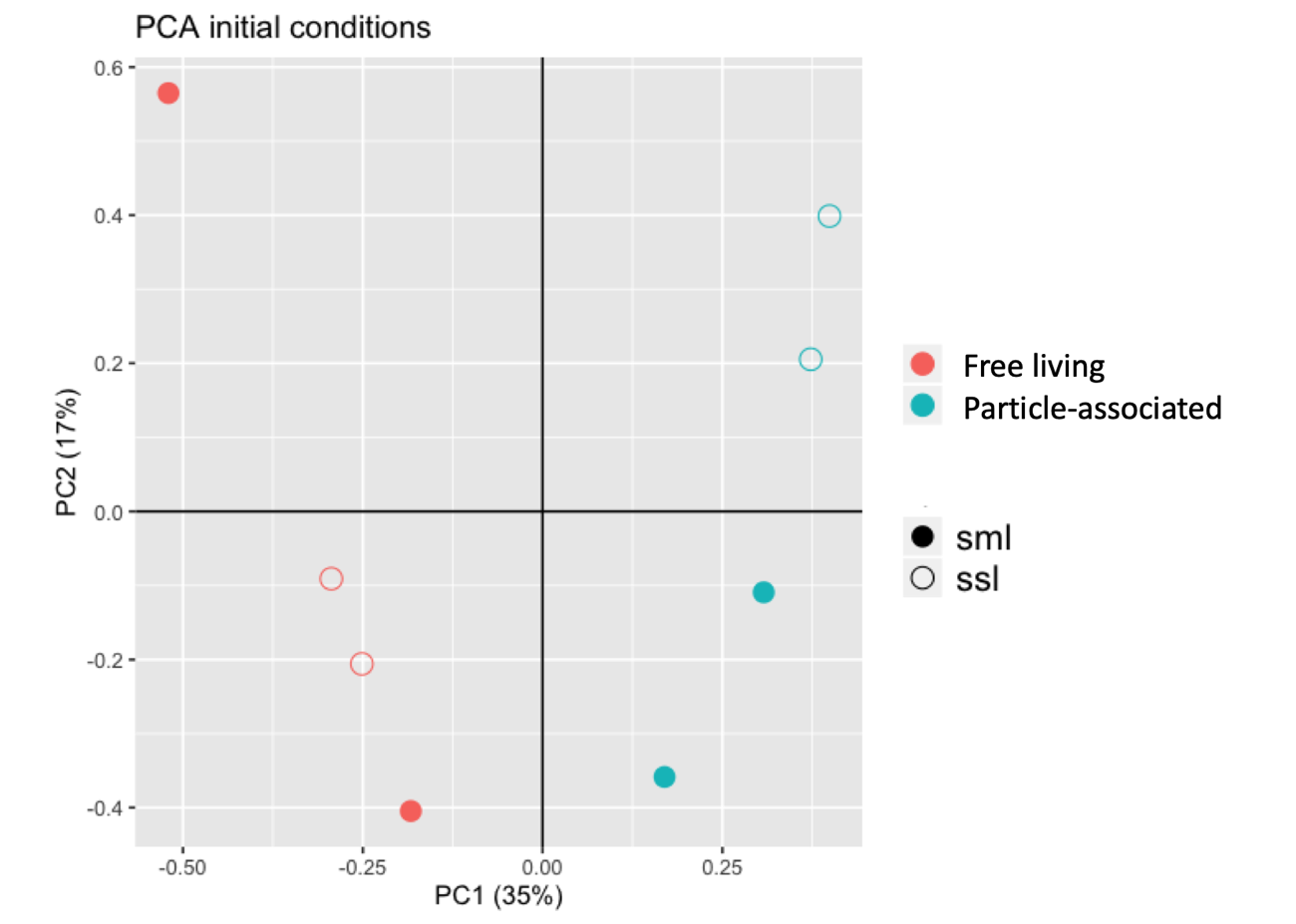


**Figure S5.** Relative abundances of hydrocarbonoclastic bacterial strains based on the 16S rRNA gene amplicon sequencing at initial conditions. Taxonomical affiliations are based on Table S6.

**Figure S6.** Relative contributions of taxa to the community composition and transcripts pool measured by 16S rRNA gene amplicon sequencing and metatranscriptomics at initial conditions and after 24 hours. The particle associated community are those bacterial cells sizing >3.0 μm, the free living fraction correspond to cells ranging from 0.2–3.0 μm. (SML: sea surface microlayer; SSL: subsurface layer; Ctrl: Control).

**Figure S7.** Fold-change in expression of genes involved in PAH degradation  between PAH treatments and controls and their taxonomic affiliation.

**Figure S8.** Fold change of the relative abundance of transcripts within SEED categories between PAH and control treatments. Significant statistical differences were detected by t-test (labeled with a *, ** or *** depending on the p-value range). (SML: sea surface microlayer; SSL: subsurface layer; PA: particle-associated; FL: free living). Different ranks in SEED categories are specified by font type: the highest rank in bold and the lowest in italics.

**Figure S9.**  Enrichment factors at the SML for PAH degradation rates. Note that Crysene and Benzo(b)flouranthene are not in the x axis since no degradation was measured at the SSL. (SML: sea surface microlayer; SSL: subsurface layer; HMW: High molecular weight; LMW: Low Molecular weight; ΣPAH: summation of compounds).


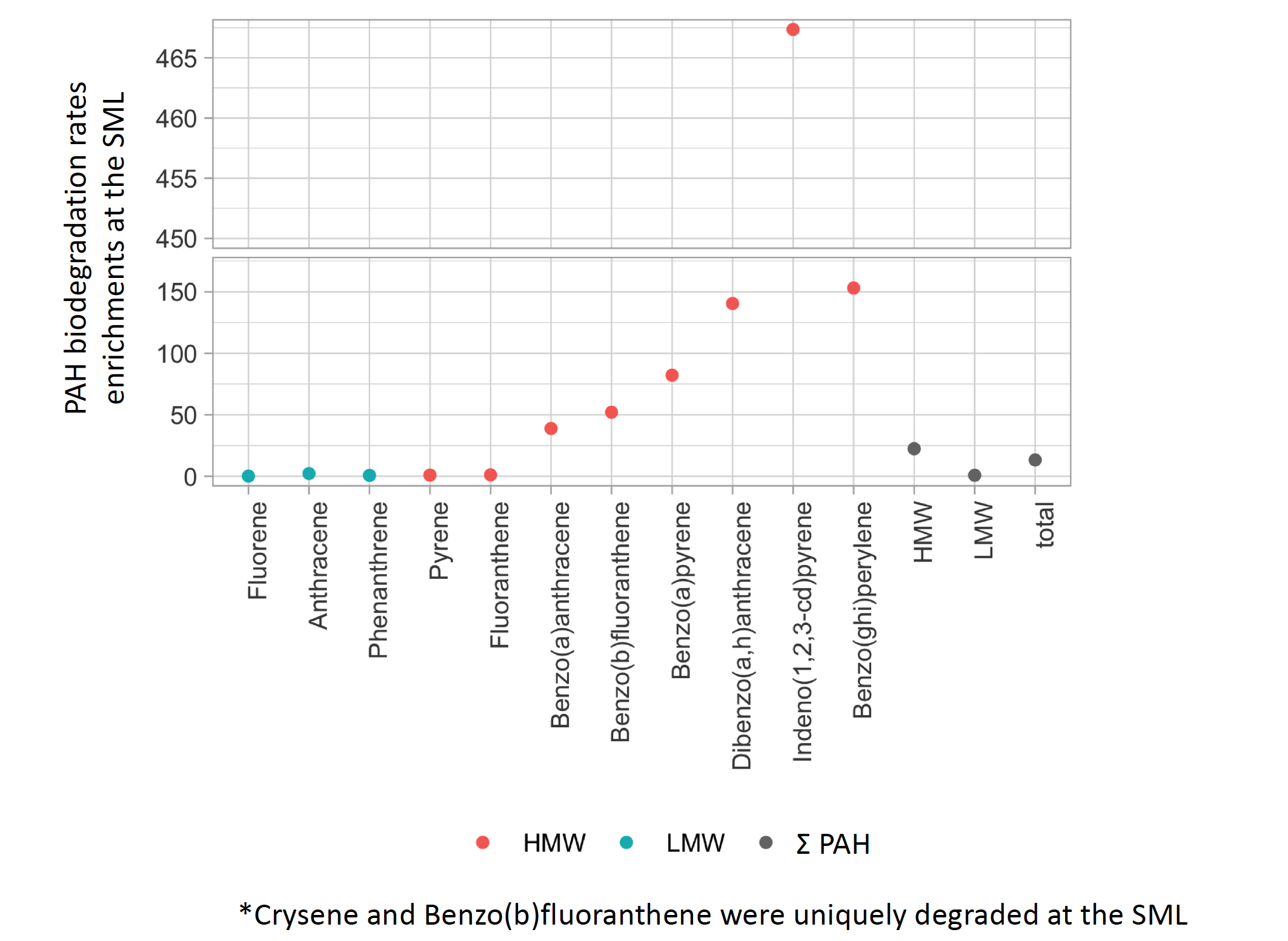


**References**

Antoniou E, Fodelianakis S, Korkakaki E, and Kalogerakis N. 2015. Biosurfactant production from marine hydrocarbon-degrading consortia and pure bacterial strains using crude oil as carbon source. *Front Microbiol* **6**.

Bodour AA, Drees KP, and Maier RM. 2003. Distribution of biosurfactant-producing bacteria in undisturbed and contaminated arid southwestern soils. *Appl Environ Microbiol* **69**: 3280–7.

Dang NP, Landfald B, and Willassen NP. 2016. Biological surface-active compounds from marine bacteria. *Environ Technol (United Kingdom)* **37**: 1151–8.

Domingues PM, Oliveira V, Serafim LS, *et al.* 2020. Biosurfactant production in sub-oxic conditions detected in hydrocarbon-degrading isolates from marine and estuarine sediments. *Int J Environ Res Public Health* **17**: 1746.

Gutierrez T, Berry D, Yang T, *et al.* 2013. Role of Bacterial Exopolysaccharides (EPS) in the Fate of the Oil Released during the Deepwater Horizon Oil Spill. *PLoS One* **8**: :e67717.

Gutiérrez T, Mulloy B, Black K, and Green DH. 2007. Glycoprotein emulsifiers from two marine Halomonas species: Chemical and physical characterization. *J Appl Microbiol* **103**: 1716–27.

Karthikeyan S, Rodriguez-R LM, Heritier-Robbins P, *et al.* 2020. Genome repository of oil systems: An interactive and searchable database that expands the catalogued diversity of crude oil-associated microbes. *Environ Microbiol* **22**: 2094–106.

Kent AG, Garcia CA, and Martiny AC. 2018. Increased biofilm formation due to high-temperature adaptation in marine Roseobacter. *Nat Microbiol* **3**: 989–95.

Lozada M, Marcos MS, Commendatore MG, *et al.* 2014. The bacterial community structure of hydrocarbon-polluted marine environments as the basis for the definition of an ecological index of hydrocarbon exposure. *Microbes Environ* **29**: 269–76.

Malavenda R, Rizzo C, Michaud L, *et al.* 2015. Biosurfactant production by Arctic and Antarctic bacteria growing on hydrocarbons. *Polar Biol* **38**: 1565–74.

Mapelli F, Scoma A, Michoud G, *et al.* 2017. Biotechnologies for Marine Oil Spill Cleanup: Indissoluble Ties with Microorganisms. *Trends Biotechnol* **35**: 860–70.

Roca C, Lehmann M, Torres CAV, *et al.* 2016. Exopolysaccharide production by a marine Pseudoalteromonas sp. strain isolated from Madeira Archipelago ocean sediments. *N Biotechnol* **33**: 460–6.

Rosenberg E, Rubinovitz C, Legmann R, and Ron E. 1988. Purification and Chemical Properties of Acinetobacter calcoaceticus A2 Biodispersan. *Appl Env Microbiol* **54**: 323–6.

Santos DKF, Rufino RD, Luna JM, *et al.* 2016. Biosurfactants: Multifunctional biomolecules of the 21st century. *Int J Mol Sci* **17**: 401.

Satpute SK, Banat IM, Dhakephalkar PK, *et al.* 2010. Biosurfactants, bioemulsifiers and exopolysaccharides from marine microorganisms. *Biotechnol Adv* **28**: 436–50.

Schulz D, Passeri A, Schmidt M, *et al.* 1991. Marine biosurfactants, I. Screening for biosurfactants among crude oil degrading marine microorganisms from the North Sea. *Z Naturforsch C J Biosci* **46**: 197–203.

Trudgeon B, Dieser M, Balasubramanian N, *et al.* 2020. Low-temperature biosurfactants from polar microbes. *Microorganisms* **8**: 1–14.

Vasileva-Tonkova E and Gesheva V. 2005. Glycolipids produced by Antarctic Nocardioides sp. during growth on n-paraffin. *Process Biochem* **40**: 2387–91.

Wittgens A, Kovacic F, Müller MM, *et al.* 2017. Novel insights into biosynthesis and uptake of rhamnolipids and their precursors. *Appl Microbiol Biotechnol* **101**: 2865–78.
